# Nobiletin Inhibits CD36-Dependent Tumor Angiogenesis, Migration, Invasion, and Sphere Formation Through the Cd36/Stat3/Nf-Κb Signaling Axis

**DOI:** 10.3390/nu10060772

**Published:** 2018-06-15

**Authors:** Nipin Sp, Dong Young Kang, Doh Hoon Kim, Jong Hwan Park, Hyo Gun Lee, Hye Jee Kim, Pramod Darvin, Yeong-Min Park, Young Mok Yang

**Affiliations:** 1 Department of Pathology, School of Medicine, Institute of Biomedical Science and Technology, Konkuk University, Seoul 05029, Korea; nipinsp@gmail.com (N.S.); kdy6459@naver.com (D.Y.K.); yorybogo@naver.com (D.H.K.); 2 Inha University College of Medicine, 27 Inhang-Ro, Jung Gu, Incheon 400-103, Korea; nihpark@yahoo.com; 3 Department of Animal Science, College of Natural Resources and Life Sciences, Pusan National University, Miryang, Gyeongsangnam 50463, Korea; ggabulzima@naver.com; 4 King’s College London GKT School of Medical Education, London SE1 1UL, UK; hye.j.kim@kcl.ac.uk; 5 Cancer Research Center, Qatar Biomedical Research Institute, Hamad Bin Khalifa University, Qatar Foundation, PO Box 5825 Doha, Qatar; promodbiotech@gmail.com; 6 Department of Immunology, School of Medicine, Konkuk University, Chungju 27478, Korea; immun3023@kku.ac.kr

**Keywords:** nobiletin, Cluster of differentiation 36 (CD36), signal transducer and activator of transcription 3 (STAT3), nuclear factor kappa-light-chain-enhancer of activated B cells (NF-κB), angiogenesis, metastasis, tumorsphere

## Abstract

Targeted cancer therapy with natural compounds is more effective than nontargeted therapy. Nobiletin is a flavonoid derived from citrus peel that has anticancer activity. Cluster of differentiation 36 (CD36) is a member of the class B scavenger receptor family that is involved in importing fatty acids into cells. CD36 plays a role in tumor angiogenesis by binding to its ligand, thrombospondin-1 (TSP-1), and then interacting with transforming growth factor beta 1 (TGFβ1). CD36 is implicated in tumor metastasis through its roles in fatty acid metabolism. This study investigated the molecular mechanisms underlying nobiletin’s anticancer activity by characterizing its interactions with CD36 as the target molecule. We hypothesize that the anti-angiogenic activity of nobiletin involving its regulation of CD36 via signal transducer and activator of transcription 3 (STAT3) rather than through TSP-1. Gene analysis identified a Gamma interferon activation site (GAS) element in the CD36 gene promoter that acts as a STAT3 binding site, an interaction that was confirmed by ChIP assay. STAT3 interacts with nuclear factor kappa-light-chain-enhancer of activated B cells (NF-κB), suggesting that nobiletin also acts through the CD36/ (STAT3)/NF-κB signaling axis. Nobiletin inhibited CD36-dependent breast cancer cell migration and invasion as well as CD36-mediated tumor sphere formation. Taken together, these results suggest that nobiletin inhibits cancer stem cells in multiple ways.

## 1. Introduction

Tumors are one of the leading cause of death worldwide. Cancer treatment is challenging, both in terms of research and in clinical medicine, and the development of anticancer agents are the primary focus of various cancer management programs. Natural compounds have been used to treat cancer for many years. The main advantage of natural compounds is that they have fewer side effects than non-natural anticancer drugs. However, natural compounds may not be target-specific; thus, it may not be possible to treat cancer using natural compounds at this time. Nonetheless, there are many natural compounds that are currently being investigated as anti-cancer agents.

**Figure 1:**
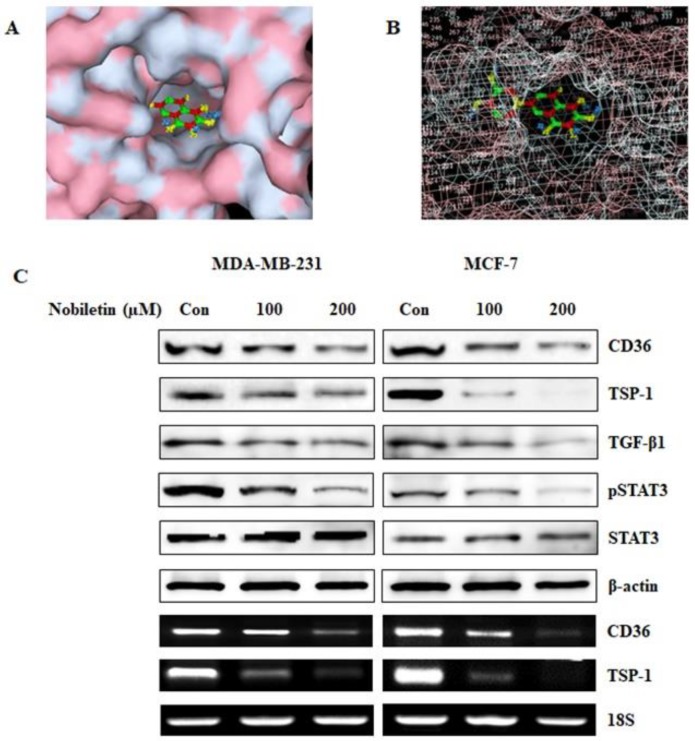
Nobiletin binds to the Cluster of differentiation 36 (CD36) receptor and inhibits its downstream targets. (**A**) The surface structure of nobiletin (PubChem ID: 72344) binding to the ATP-binding domain of CD36 (PDB ID: 5LGD) as determined by molecular docking using Autodock Vina software. (**B**) The mesh structure of nobiletin-CD36 binding obtained using PyMol software. (**C**) Western blotting and Reverse transcription polymerase chain reaction (RT-PCR) analysis of MCF-7 and MDA-MB-231 cells showing that nobiletin treatment for 24 h inhibits the downstream targets of CD36 in a concentration-dependent manner.

Nobiletin (5,6,7,8,3,4′-hexamethoxyflavone) is a flavonoid that is isolated from citrus peel (Figure 1A). This anti-inflammatory drug can suppress cartilage degradation [1], enhance α-amino-3-hydroxy-5-methyl-4-isoxazolepropionic acid (AMPA) receptor activity [2], and regulate matrix metalloproteinases (MMPs) [3]. Further, nobiletin inhibits some of cancer hallmarks [4,5,6] and the activity of aromatase, an enzyme that is an important target in cancer cells [7]. A recent study reported that nobiletin inhibits angiogenesis by regulating Src/FAK/STAT3-mediated signaling through PXN in ER^+^ breast cancer cells [8].

In humans, cluster of differentiation 36 (CD36) is encoded by the CD36 gene. CD36 imports fatty acids into cells and is a member of the class B scavenger receptor family of cell surface proteins. CD36 binds to various ligands, including thrombospondin-1 (TSP-1) [9], and thus has a role in the regulation of angiogenesis. Anti-angiogenic drugs represent a potential therapeutic strategy for controlling the spread of cancer [10]. Some studies suggest that fatty acid uptake via CD36 may promote cancer cell migration and proliferation. Specifically, CD36 regulates apoptosis and angiogenesis in response to its ligand, TSP-1 [11]. Blocking TSP1 from binding to its cell surface receptor makes normal tissue resistant to cancer radiation therapy and thereby initiates tumor cell death [12]. TSP-1 binds to CD36 and then to transforming growth factor beta 1 (TGFβ1), inhibiting growth factor-induced pro-angiogenic signals that mediate endothelial cell proliferation, tube formation, and migration [13]. Recent research suggests that CD36 interacts with the STAT3 (signal transducer and activator of transcription 3) oncogene [14]. STAT3 then interacts with nuclear factor kappa-light-chain-enhancer of activated B cells (NF-κB) in the nucleus, leading to carcinogenesis [15].

Recent research shows that CD36 plays a role in tumor metastasis by mediating lipid metabolism. Overexpression of CD36 (CD36^+^) increases fatty acid uptake, significantly increasing the rate of lymph node metastasis, while the absence of CD36 decreases tumor metastasis. CD36^+^ cells are highly metastatic, and treatment with anti-CD36 neutralizing agents may inhibit the initiation of metastasis [16]. Studies have reported that patients with advanced stage ovarian cancer showed upregulated CD36 expression and that inhibition of CD36 reduced tumor growth and metastasis [17]. Notably, fatty acids also play a vital role in CD36^+^-associated metastases, suggesting that CD36^+^ promotes metastasis by regulating β-oxidation and fatty acids. The enzyme 3-hydroxy-3-methylglutaryl-CoA synthase 2 (HMGCS2) plays a vital role in the β-oxidation pathway [18]. Accordingly, an anticancer drug that can target CD36, TSP-1, and HMGCS2 during tumor treatment has great potential for slowing or blocking tumor metastasis. 

## 2. Results

### 2.1. Nobiletin Binds to CD36 and Inhibits the Expression of CD36 and its Downstream Target Proteins

CD36 acts as a fatty acid receptor and plays a key role in tumor metastasis by regulating lipid metabolism. Thus, we investigated nobiletin binding to CD36. Molecular docking results showed that nobiletin binds to CD36 (Figure 1B). Specifically, atoms 11 to 20 bound to the extracellular domain of the CD36 protein (Figure 1C), confirming that nobiletin binds to CD36. We then analyzed the downstream targets of CD36 after the addition of nobiletin at various concentrations. Translational and transcriptional analysis showed that 200 µM nobiletin inhibited CD36 expression and its binding domain TSP-1 in a concentration-dependent manner (Figure 1D). Nobiletin also decreased the expression of phosphorylated STAT3, which plays an important role in angiogenesis. These results may suggest that nobiletin regulates CD36 through STAT3 rather than through TSP-1.

### 2.2. Nobiletin Inhibits CD36-Mediated In Vitro Angiogenesis by Regulating STAT3

**Figure 2:**
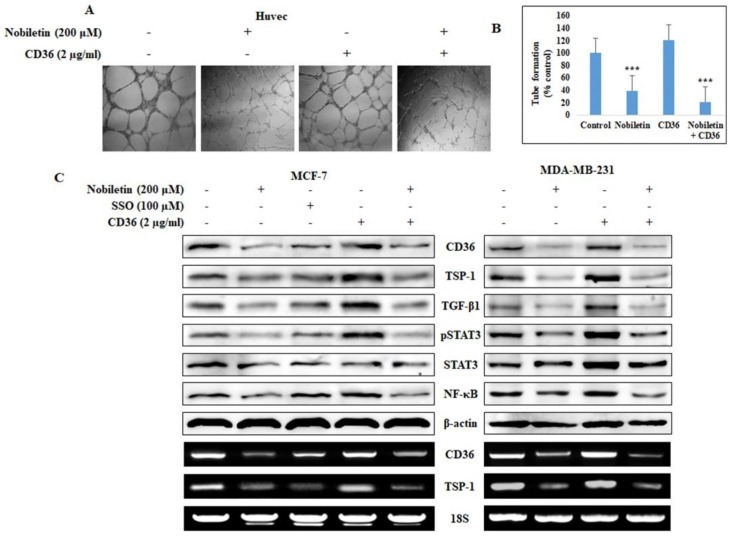
Nobiletin inhibits CD36-dependent angiogenesis and regulates STAT3 expression in vitro. (**A**) Nobiletin inhibits CD36-dependent angiogenesis in HUVEC cells in an in vitro angiogenesis assay. Recombinant human CD36 (2 µg/mL) was used to induce CD36 expression. (**B**) Graph of the results of the in vitro angiogenesis assay showing the relative inhibition of CD36-mediated angiogenesis. Statistical analysis was performed using Analysis of variance (ANOVA) (*** *p* < 0.001). (**C**) Western blotting and RT-PCR analysis in MCF-7 cells showing the CD36-mediated inhibition of downstream targets of CD36. SSO (100 µM) was used as a CD36 inhibitor.

Nobiletin inhibits angiogenesis in endothelial cells. Accordingly, we investigated the effects of recombinant human CD36 in angiogenesis suppression in Human Umbilical Vein Endothelial Cells (HUVEC) cells treated with 200 µM nobiletin (Figure 2A). Compared to control cells, HUVEC cells that were treated with CD36 showed a slight increase in tube formation, and nobiletin significantly inhibited this increase (Figure 2B). We then analyzed the effects of nobiletin on the CD36-mediated expression of downstream target proteins. We observed an increase in the expression of these proteins in MCF-7 and MDA-MB-231 cells that were treated with recombinant human CD36, and this increase was inhibited by nobiletin (Figure 2C). Sulfo-N-succinimidyl oleate (SSO; 100 µM) is an inhibitor of CD36. CD36-treated cells showed increased STAT3 and NF-κB expression, and treatment with 200 µM nobiletin mitigated this effect. This showed the relationship of CD36 with STAT3 and NF-κB.

### 2.3. Nobiletin Inhibits the Nuclear Translocation of STAT3 and Its Binding to the CD36 Gene Promoter

**Figure 3:**
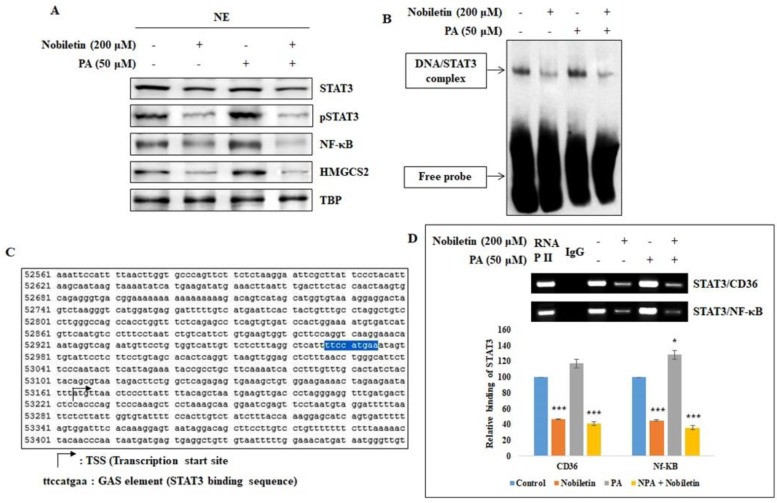
Nobiletin inhibits the CD36-dependent binding of STAT3 to DNA and to the gene promoter. (**A**) Western blotting analysis of nuclear extracts from cells treated with nobiletin and palmitic acid showing the regulation of nuclear translocation of STAT3, NF-κB, and HMGCS2. (**B**) Electrophoretic mobility shift assay (EMSA) analysis showing that palmitic acid enhances STAT3 binding to the Gamma interferon activation site (GAS) element and that nobiletin inhibits this binding. (**C**) Sequence of the human CD36 gene promoter (https://www.ncbi.nlm.nih.gov/nuccore/NG_008192.1?from=5001&to=77233&report=genbank). A GAS element (TTCCATGAA) in the CD36 gene (nucleotide sequence 52967-52975) is highlighted. (**D**) ChIP assay analysis shows that palmitic acid enhances the formation of the STAT3/CD36 and STAT3/(NF-κB) complexes while nobiletin inhibits their formation. The relative binding of STAT3 to the CD36 gene and to the NF-κB gene promoter is expressed as a percentage of control. Statistical analysis was performed using ANOVA (*** *p* < 0.001). * denotes the statistical significance of the result which obtained by performing ANOVA test.

Since CD36-dependent angiogenesis was inhibited by nobiletin, we investigated the role of CD36 in the nuclear translocation of the STAT3 oncogene. We assumed that STAT3 plays a vital role in CD36 activity, and we used a fatty acid, palmitic acid (PA), which binds to CD36 and enhances its expression, to investigate this role. Western blotting analysis of nuclear extracts from MCF-7 cells showed an increase in STAT3 expression in PA-treated cells that was reversed when nobiletin was added to the PA-treated cells (Figure 3A). Nobiletin also decreased the expression of HMGCS2, which is a key enzyme in beta oxidation. Next, we analyzed the binding of STAT3 to the CD36 gene promoter using an electrophoretic mobility shift assay (EMSA). We observed increased STAT3 DNA binding activity in PA-treated cells that was inhibited by nobiletin, demonstrating that STAT3 plays a direct role in CD36 expression (Figure 3B).

To further investigate the underlying mechanism, we searched the CD36 gene for a GAS element, which is considered to be a STAT3 binding domain. We found a GAS element (TTCCATGAA) in the CD36 gene promoter region (nucleotides 52967–52975) that seemed likely to be a STAT3 binding site (Figure 3C). ChIP assay results confirmed the binding of STAT3 to the CD36 gene as the STAT3 binds to the GAS element in the CD36 gene promoter (Figure 3D). There was an increase in the formation of the STAT3/CD36 complex in PA-treated cells, confirming the relationship between STAT3 and CD36. We then analyzed whether nobiletin affected the binding of STAT3 to the NF-κB promoter region, which is a well-known downstream target of STAT3. Taken together, these results suggested that CD36 acts through the STAT3/NF-κB signaling axis.

### 2.4. Nobiletin Suppresses STAT3 Expression in a CD36-Dependent Manner

**Figure 4:**
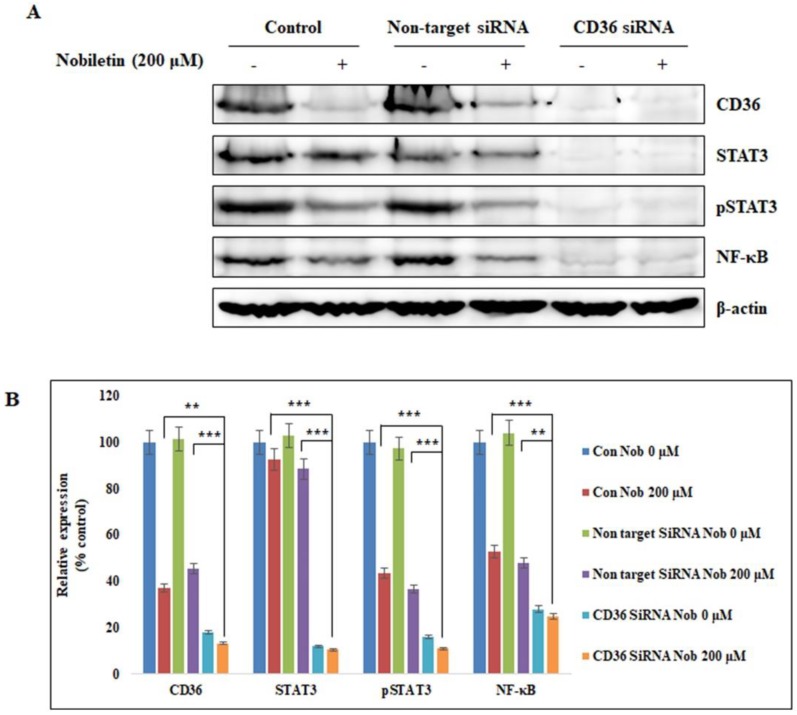
Nobiletin suppresses STAT3 expression in a CD36-dependent manner. (**A**) Western blotting analysis of the inhibition of CD36 expression by ON-TARGET*plus* CD36 siRNA as well as the expression of CD36, STAT3, and NF-κB in MCF-7 cells after treatment with nobiletin. (**B**) The relative expression of the CD36, STAT3, pSTAT3, and NF-κB proteins in MCF-7 cells. Statistical analysis was performed using the Student’s *t*-test (** *p* < 0.01, *** *p* < 0.001).

After we found that STAT3 binds to CD36, we investigated the effects of STAT3 on CD36 activity by using siRNA to silence the CD36 gene in MCF-7 cells. We knocked down the expression of CD36 with specific ON-TARGET*plus* human CD36 siRNA (Dharmacon) followed by treatment with 200 µM nobiletin for 24 h. Then we isolated the proteins and analyzed the expression levels of key proteins using Western blotting. Notably, CD36 expression was completely inhibited in CD36 siRNA-treated cells (Figure 4A). STAT3 and NF-κB expression also decreased in CD36 siRNA-treated cells. Nobiletin inhibited the expression of phospho-STAT3 and NF-κB, demonstrating the involvement of the CD36/STAT3/NF-κB signaling axis (Figure 4B).

### 2.5. Nobiletin Inhibits CD36-Dependent Breast Cancer Cell Migration and Invasion

**Figure 5:**
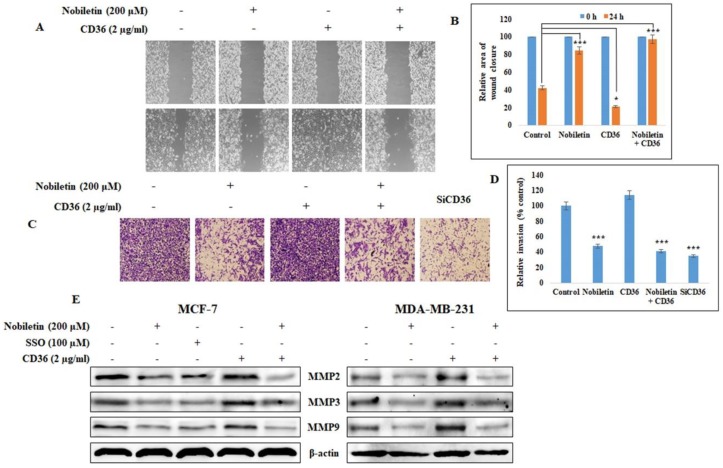
Nobiletin inhibits CD36-dependent cell migration, invasion, and matrix metalloproteinases (MMP) expression. (**A**) MDA-MB-231 cell migration was inhibited by 24-h treatment with nobiletin and recombinant human CD36 in a wound-healing assay. (**B**) The relative wound closure area in the presence of CD36 and nobiletin. Statistical analysis was performed using ANOVA (* *p* < 0.05, *** *p* < 0.001). (**C**) The Matrigel cell invasion assay shows that MDA-MB-231 cell invasion is enhanced by recombinant CD36 and inhibited by 24-h treatment with nobiletin. (**D**) The relative invasion of MDA-MB-231 cells after treatment with recombinant CD36 and nobiletin. Statistical analysis was performed using ANOVA (*** *p* < 0.001). **E**) Western blotting analysis of CD36-dependent MMP expression and its inhibition by nobiletin in MDA-MB-231 and MCF-7 cells.

CD36 is considered to be a major factor in tumor metastasis. To validate this, we analyzed tumor cell migration and invasion, which are the two key processes in tumor metastasis. A wound healing assay showed that treatment with recombinant human CD36 protein induced almost complete wound closure after 24 h (Figure 5A), but the addition of nobiletin significantly inhibited wound closure. This suggests that CD36 plays a role in migration (Figure 5B). Invasive potential was determined using the Matrigel invasion assay (Figure 5C). These results suggested that CD36 also plays a role in invasion, since treatment with recombinant human CD36 protein increased invasion and treatment with CD36 siRNA suppressed invasion. Nobiletin significantly decreased the CD36-dependent invasive potential of the cells (Figure 5D). To confirm CD36-mediated invasion, we analyzed the expression of MMPs, since invasion depends upon the release of MMPs. Figure 5E shows that nobiletin inhibited the CD36-dependent expression of MMP2, MMP3, and MMP9. These results show that CD36 plays a role in tumor metastasis and that nobiletin inhibits CD36-mediated tumor metastasis.

### 2.6. Nobiletin Inhibits CD36-Mediated Tumorsphere Formation and Survival

**Figure 6:**
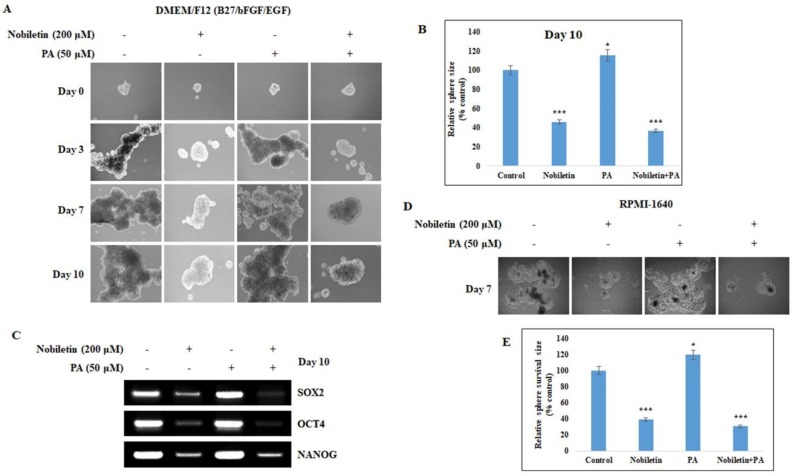
Nobiletin inhibits CD36-dependent tumor sphere formation and tumor sphere survival. (**A**) Sphere formation was enhanced by palmitic acid treatment and inhibited by nobiletin treatment after MCF-7 cells were cultured in DMEM/F12 media containing EGF, bFGF, and B27 for 10 days. (**B**) Graph showing tumor sphere size after 10 days. Statistical analysis was performed using ANOVA (* *p* < 0.05, *** *p* < 0.001). (**C**) Reverse transcription polymerase chain reaction (RT-PCR) analysis of stem cell markers showing their upregulation in response to palmitic acid and their downregulation in response to treatment with nobiletin. (**D**) Tumor sphere survival analysis was performed by seeding the tumor spheres in RPMI-1640 cancer cell media for 7 days without palmitic acid or nobiletin. (**E**) Graph showing tumor sphere survival after 7 days without further drug treatment. Statistical analysis was performed using ANOVA (* *p* < 0.05, *** *p* < 0.001).

We showed that CD36 has a role in tumor migration and invasion, which are key for metastasis. Next, we investigated the role of CD36 in tumor sphere formation. The tumor sphere assay was performed by seeding MCF-7 cells in Dulbecco’s Modified Eagle Medium/Nutrient Mixture F-12 (DMEM/F12) media containing the growth supplements Epidermal growth factor (EGF), basic fibroblast growth factor (bFGF), and B27. After 10 days of incubation, there was an increase in the size of tumor spheres in PA-treated cells that was mitigated by nobiletin (Figure 6A). There was a significant increase in sphere size in PA-treated cells, and nobiletin inhibition of tumor sphere formation was highly significant (Figure 6B). In order to confirm tumor sphere inhibition by nobiletin, we isolated RNA from the spheres and determine the expression of the SOX2, OCT4, and NANOG genes, which are considered stem cell markers. We found that nobiletin inhibited the expression of these tumor marker genes (Figure 6C), suggesting that nobiletin inhibited CD36-mediated tumor sphere formation. To investigate tumor sphere survival, we reseeded the tumor spheres in RPMI-1640 cancer cell media and incubated them for 7 days without PA or nobiletin (Figure 6D). Control and PA-treated spheres showed significantly higher sphere survival compared to nobiletin-treated spheres, which did not survive in RPMI-1640 media (Figure 6E). This suggested that nobiletin has prolonged anti-tumor effects.

### 2.7. Nobiletin Inhibits CD36-Mediated Fatty Acid-Regulated Proteins

**Figure 7:**
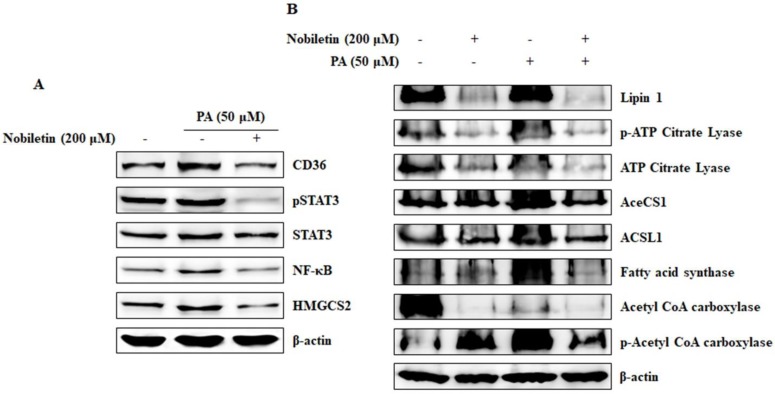
Nobiletin inhibits fatty acid-induced CD36 expression and expression of the downstream targets of CD36. (**A**) Western blotting analysis shows the enhancement of CD36 expression and its inhibition by nobiletin. It also shows that nobiletin inhibits the expression of the beta-oxidation enzyme 3-hydroxy-3-methylglutaryl-CoA synthase 2 (HMGCS2). (**B**) Western blotting analysis of proteins involved in fatty acid and lipid metabolism (Lipin1, ATP-citrate lyase, AceSC1, ACSL1, fatty acid synthase, and ACC) in the presence of 50 μM palmitic acid and 200 μM nobiletin.

We showed that CD36 can regulate tumor metastasis and tumor sphere formation in cells treated with PA. Here we analyzed the signaling CD36/STAT3/NF-κB axis along with HMGCS2. The results showed that nobiletin inhibited the PA-induced expression of CD36, phospho-STAT3, NF-κB, and HMGCS2 (Figure 7A). To investigate the roles of fatty acids in CD36 expression, we analyzed the expression of proteins involved in fatty acid and lipid metabolism in the presence of PA and nobiletin (Figure 7B). The results suggested that PA induced CD36 expression as well as the expression of Lipin1, activated ATP citrate lyase, fatty acid synthase, and activated acetyl Co-A carboxylase (ACC). In contrast, acetyl-CoA synthetase-1 (AceCS1) and acyl-CoA synthetase long-chain family member-1 (ACSL1) had no role in PA-induced CD36 expression.

## 3. Discussion

Anticancer treatment with naturally-derived agents is a good approach in terms of reducing the burden of side effects [19]. However, naturally-derived drugs lack specific targets, which is a disadvantage of this strategy. Targeted treatment with natural products would be more efficacious. Here we investigated a natural flavonoid, nobiletin, that acts as an anti-cancer agent against various cancers [20,21,22]. However, the molecular mechanisms underlying the effects of nobiletin remain unclear.

CD36 is a basic marker for cancer cells that plays important roles in tumor angiogenesis and tumor metastasis. It interacts with TSP-1 and then with Transforming growth factor beta 1 (TGF-β1) to promote tumor angiogenesis [23]. Here we found that nobiletin inhibited angiogenesis by suppressing CD36 expression and decreasing the expression of TSP-1 and TGF-β1. Notably, TSP-1 is considered an endogenous inhibitor of angiogenesis [24]. This suggested that the inhibition of angiogenesis by nobiletin was independent of TSP-1 and TGF-β1; indeed, we hypothesized that nobiletin regulated CD36 through STAT3 signaling (Figure 1D and Figure 2C). These results suggested an interaction between CD36 and STAT3 in the presence of nobiletin. When we analyzed the binding of STAT3 to CD36, we found that STAT3 could bind to the GAS element of the CD36 promoter [25]. We identified a GAS element (TTCCATGAA) in the CD36 gene (Figure 3C), suggesting that STAT3 binds to that GAS element. We confirmed the binding of STAT3 to CD36 using a ChIP assay. We designed a primer that encoded the GAS element in the CD36 gene and confirmed its binding by STAT3/CD36 complex formation (Figure 3D). It seemed likely that STAT3 then interacted with NF-κB to promote transcription [15], and we observed STAT3/NF-κB binding, which was inhibited by nobiletin. Taken together, these results suggested that nobiletin’s effects are mediated by the CD36, STAT3, and NF-κB signaling proteins. We confirmed the interaction between CD36 and STAT3 using CD36 siRNA (Figure 4). The inhibition of NF-κB expression by nobiletin also supported the idea that nobiletin acts through the CD36/STAT3/NF-κB signaling axis.

Recent studies suggest that CD36 overexpression induces tumor metastasis by affecting lipid metabolism [16]. Tumor migration and invasion are critical for tumor metastasis. Accordingly, we analyzed the role of CD36 in tumor migration and invasion and found that nobiletin inhibited CD36-dependent tumor migration and invasion. Using the MDA-MB-231 cell line, we found that the addition of recombinant human CD36 increased cell migration by 20% and cell invasion by 16% compared to control cells (Figure 5B,D). Treatment with CD36 siRNA inhibited cell invasion by almost 65%, suggesting that CD36 plays a role in tumor metastasis. These results were confirmed by expression analysis of MMPs, which are key proteins in tumor migration and invasion [26].

Tumor sphere-based assays are considered efficient in vitro platforms for cancer stem cell (CSC) studies, as tumor spheres have all of the characteristics of tumor stem cells [27]. CSCs are a sub-population of cancer cells that have tumor initiation properties and a tendency to form bulk cells [28]. For tumor sphere formation, we seeded MCF-7 cells in DMEM/F12 media containing the growth supplements EGF, bFGF, and B27 in low-attachment six-well plates. This led to the selective growth of a tumor sphere. We observed bulk tumor cells in the wells of control cells and PA-treated cells that showed tumor sphere characteristics; notably, their formation was inhibited by nobiletin (Figure 6A). To confirm tumor sphere formation, we analyzed the expression levels of SOX2, OCT4, and NANOG, which are considered to be stem cell markers [29]. Nobiletin inhibited the expression of stem cell markers, suggesting its ability to inhibit cancer stem cell growth. Fatty acids regulate CD36 expression through beta oxidation and fatty acid metabolism, and HMGCS2 is an important enzyme in beta oxidation [18]. HMGCS2 also played a role in the inhibition of CD36 by nobiletin (Figure 3A and Figure 7A). Some proteins that are involved in fatty acid metabolism are also involved in CD36-mediated tumor-promoting activities that are inhibited by nobiletin. The major proteins involved in fatty acid and lipid metabolism are Lipin1 [30], ATP-citrate lyase [31], AceSC1 [32], ACSL1 [33], fatty acid synthase [34], and ACC [35]. We found that PA induced CD36-enhanced expression of these proteins and their phosphorylated forms, except for AceCS1 and ACSL1. These results suggested that these proteins may also affect tumor metastasis through the CD36 receptor.

In conclusion, nobiletin inhibited CD36-mediated in vitro angiogenesis through STAT3. STAT3 interacted with CD36, which then interacted with NF-κB; thus, nobiletin inhibited tumor angiogenesis via the CD36/STAT3/NF-κB signaling axis. Nobiletin also inhibited CD36-dependent tumor migration, invasion, and tumor sphere formation.

## 4. Materials and Methods

### 4.1. Antibodies and Reagents

Human breast adenocarcinoma, MCF-7, and MDA-MB-231 cell lines were purchased from South Korean Cell Bank (Seoul, Korea). RPMI-1640, DMEM/F12 β-actin antibody and Imprint chromatin immunoprecipitation assay kit was purchased from Sigma Chemical (St. Louis, MO, USA). Penicillin-streptomycin solution and fetal bovine serum (FBS) were purchased from Hyclone (South of Logan, Utah). 0.05% trypsin-ethylenediaminetetraacetic acid was purchased from Gibco-BRL (Grand Island, NY, USA). In vitro angiogenesis kit and was purchased from Millipore (Billerica, MA, USA). CD36, TSP-1, TGFβ1, STAT3, p-STAT3, MMP2, MMP3, MMP9 antibodies, and secondary antibodies (goat anti-mouse and rabbit immunoglobulin G [IgG]-horseradish peroxidase) were obtained from Santa Cruz Biotechnology (Santa Cruz, CA, USA). Anti-HMGCS2, and anti-TBP antibodies were purchased from Abcam (Cambridge, UK). Anti- NF-κB antibody was purchased from Cell Signaling Technology (Beverly, MA, USA). The WesternBright ECL HRP substrate detection solution was purchased from Advansta Inc. (Menlo Park, CA, USA). Restore™ Western Blot Stripping Buffer and NE-PER kits were purchased from Pierce (Rockford, IL, USA). RNeasy mini kits and QIAprep Spin Miniprep Kits were purchased from QIAGEN (Hilden, Germany). Reverse transcriptase-polymerase chain reaction (RT-PCR) premix kits and CD36, TSP-1, NF-κB, SOX2, OCT4, NANOG and 18s primers for RT-PCR were synthesized by Bioneer (Daejon, Korea). Electrophoretic mobility shift assay (EMSA) kit and oligonucleotide probes (STAT3) were obtained from Promega Corp. (Madison, WI, USA).

### 4.2. Molecular Docking

Molecular docking was used to find the binding ability of nobiletin to CD36 receptor. It was done by using PyRx software in an autodock vina platform. 3D structure of nobiletin obtained from PubChem (ID: 72344) and 3D structure of CD36 obtained from Protein Data Bank (PDB ID: 5LGD). The obtained of docked products were visualized by PyMol software.

### 4.3. Cell Culture and Treatment

ER^+^ cell line, MCF-7 cell line and triple negative cell line, MDA-MB-231 were maintained in RPMI-1640 medium containing 10% FBS, 100 U/mL penicillin at 37 °C in 5% CO_2_. Endothelial cell line, HUVEC was maintained in EBM-2 endothelial growth basal media. The cells were placed in airtight chambers (Nu Aire, Plymouth, MN, USA). At the beginning of each experiment, the cells were counted depends on experiments and re-suspended in the medium. Cells were treated with nobiletin at different concentration according to the experiments. Media for tumor sphere formation was made by adding the growth supplements B27, EGF and bFGF to DMEM/F12 media.

### 4.4. *In vitro* Angiogenesis Assay

ECMatrix were thawed at 4 °C for overnight, and required wells of pre-chilled 96-well plates were coated with diluted EC Matrix of 50 μL and incubated at 37 °C for 1 h to solidify. 150 μL of HUVEC cells (1 × 10^4^) with or without nobiletin, recombinant CD36 protein and Sulfo-*N*-succinimidyl oleate (SSO) were added to the solidified matrix and incubated at 37 °C for 12 h. Endothelial cell formation was observed using a microscope. Focus was placed on distinct areas and the tubes formed were counted according to the kit procedure.

### 4.5. Western Blotting

The MCF-7 and MDA-MB-231 cell lines were treated with nobiletin, recombinant CD36 protein, SSO or palmitic acid for 24 h. Whole cells were lysed on ice with radio immunoprecipitation lysis buffer containing protease and phosphatase inhibitors. Cells were scrapped out and centrifuged at 15,000 rpm for 10 min at 4 °C to remove cellular debris. Protein concentrations were measured using the Bradford method. Equal amounts of proteins were resolved on sodium dodecyl sulfate-polyacrylamide (SDS) gel electrophoresis (SDS-PAGE) and transferred onto nitrocellulose membrane. The blots were blocked for 1 h with 5% BSA (Bovine serum albumin). Membranes were probed over night at 4 °C with a primary antibody followed by HRP-conjugated secondary antibodies. Detection was performed using the ECL Plus detection kit and an LAS-4000 imaging device (Fujifilm, Japan).

### 4.6. Reverse Transcription-PCR

Total RNAs were extracted using RNeasy Mini Kits (QIAGEN) and quantified spectrometrically at 260 nm. RT-PCR analysis for CD36, TSP-1, SOX2, OCT4, NANOG and 18 s RNAs were then performed. cDNA was synthesized from total RNA by RT at 42 °C for 1 h and 80 °C for 15 min using first strand cDNA synthesis kits (Bioneer, Korea). PCR was conducted using cDNA. The PCR conditions consisted of denaturation for 30 s–1 min at 94–95 °C, annealing for 45 s–1 min at 55–58 °C, and extension for 45 s–1 min at 72 °C for 30 cycles. The primers used for the amplification are listed in the Table 1. PCR products were analyzed by 1% agarose gel stained with ethidium bromide.

### 4.7. EMSA

The DNA binding activity of STAT3 was assessed using EMSA, in which a labeled double-stranded DNA was used as a DNA probe to bind active STAT3 proteins in the nuclear extracts. Nuclear protein extracts were isolated with a nuclear extract kit (Panomics, AY2002). The EMSA experiment was conducted by incubating a biotin-labeled transcription factor-STAT3 probe with treated and untreated nuclear extracts. Proteins were resolved in a nondenaturing 6% PAGE gel (Bio-Rad, Korea). The proteins in the gel, transferred to a nylon membrane and then detected using streptavidin-HRP and a chemiluminescent substrate in a LAS-4000 imaging device.

### 4.8. ChIP Assay

A ChIP assay was performed using an Imprint Chromatin Immunoprecipitation Kit according to the manufactures protocol. MDA-MB-231 cells were fixed with 1% formaldehyde and quenched with 1.25 M glycine. Then the cells were suspended in nuclei preparation buffer and sonicated in shearing buffer under optimized conditions. This sheared DNA was diluted with dilution buffer (1:1 ratio). The diluted supernatant was incubated with antibody (STAT3) in pre-coated wells for 90 min. Normal mouse IgG and anti-RNA polymerase II were used for negative and positive control. The unbound DNA was washed off with IP wash buffer and the bound DNA was collected by cross link reversal using DNA release buffer containing proteinase K. The released DNAs and the DNA from the internal controls were purified with GenElute Binding Column G. The DNA was then quantified using specific CD36 and NF-κB primers (Table 1) by conventional PCR.

### 4.9. Wound Healing Assay

MDA-MB-231 cells were cultured in six-well plates at 1 × 10^5^ cells/well in RPMI-1640 media and incubated for 24 h. After becoming a confluent monolayer, the cell layers were scratched with a pipette tip and washed with PBS to remove cell debris. Cells were treated with recombinant human CD36 protein and nobiletin. Control cells were not treated. Wound edges were photographed at different time intervals using a microscope. The relative area of wound closure was measured using ImageJ software (NIH Image, Bethesda, MD, USA).

### 4.10. Matrigel Invasion Assay

The transwell invasion assay was conducted with the help of Matrigel pre-coated, ready to use invasion chambers (BD Biocoat, MA, USA). 5 × 10^4^ cells were added to the inserts. The drug-containing media was added to the receiver plate and the inserts containing cells were placed onto it. After a 24 h incubation in a humidified chamber at 37 °C, the cells that invaded to the apical surface of the inserts were resolved with crystal violet. The cells on the upper surface were removed using a cotton swab and the invaded cells were observed using a microscope. Focus was placed on four distinct areas and the cells were counted.

### 4.11. Tumor Sphere Formation Assay

Tumor sphere assay was performed by culturing MCF-7 cells in DMEM/F12 media containing growth supplements, EGF, bFGF and B27 in Low attachment 6 well plates. The treatment day was considered as day 0 and incubated the cells for 10 days. Photographs were taken at day 3, 7 and 10 using microscope. Total RNA was isolated from the sphere and analyzed using RTPCR. Tumor sphere survival was done by reseeding the tumor spheres to the RPMI-1640 and incubated for 10 days without containing any drugs. Photographs were taken at the end of day 10.

### 4.12. Small Interference RNA (siRNA) Analysis

MCF-7 cells (1 × 10^5^) were cultured on 6-well plates and grown to 60% confluence. The cells were then transfected with ON-TARGET plus SMARTpool siRNA targeting CD36 or ON-TARGETplus non-targeting siRNA using DharmaFECT transfection reagent (Dharmacon, Chicago, IL, USA) according to the manufacturer’s instructions. Following transfection with this mixture for 24 h, nobiletin treated for further 24 h. The proteins were isolated and resolved in a SDS-PAGE to check the expression pattern.

### 4.13. Statistical Analysis

All the experiments were repeated three times and the results were expressed as mean ± SEM. Groups were compared with the *ANOVA*-test or Student’s *t*-test. Statistical analyses were performed with the SAS program.

## 5. Conclusions

These studies showed that nobiletin bound to CD36 in breast cancer cells and thereby inhibited tumor angiogenesis and tumor metastasis. This inhibition was regulated by the CD36/STAT3/NF-κB signaling axis. Nobiletin also inhibited CD36-mediated tumor sphere formation. 

## Figures and Tables

**Table 1:** RT-PCR primer sequences, annealing temperature and product sizes.

Sl No	Gene	Annealing Temperature (°C)	Product Size (bp)	Sequence (5′–3′)
**1**	CD36	55	768	F-ggcaccactgtgtacagacag R-ggaaaggaggctgcgtctgtgc
**2**	TSP-1	60	345	F-gttgcatgtgtgtggaagcaac R-accacactgaagatctggccag
**3**	18S	58	490	F-agccttcggctgactggctgg R-ctgcccatcatcatgacctgg
**4**	SOX2	55	165	F-ctgcagtacaactccatgac R-gagtgggaggaagaggtaac
**5**	OCT4	55	190	F-actggttcgctttctctttc R-aaggtattcagccaaacgac
**6**	NANOG	55	202	F-ctcctccatggatctgctta R-ggctgaggtatttctgtctc
**7**	CD36 (ChIP assay)	55	191	F-ttctcagagcctcagtgtga R-acctgagtgtgctacaggaa
**8**	NF-κB (ChIP assay)	55	123	F-tcctacctgaaggcaaagga R-ccgtttcatagaaagggcca
